# Interaction of brain 5-HT synthesis deficiency, chronic stress and sex differentially impact emotional behavior in Tph2 knockout mice

**DOI:** 10.1007/s00213-015-3879-0

**Published:** 2015-02-27

**Authors:** Lise Gutknecht, Sandy Popp, Jonas Waider, Frank M. J. Sommerlandt, Corinna Göppner, Antonia Post, Andreas Reif, Daniel van den Hove, Tatyana Strekalova, Angelika Schmitt, Maria B. N. Colaςo, Claudia Sommer, Rupert Palme, Klaus-Peter Lesch

**Affiliations:** 1 Division of Molecular Psychiatry, Laboratory of Translational Neuroscience, Department of Psychiatry, Psychosomatics and Psychotherapy, University of Wuerzburg, Wuerzburg, Germany; 2 Department of Psychiatry, Psychosomatics and Psychotherapy, University of Wuerzburg, Wuerzburg, Germany; 3 Department of Biomedical Sciences/Physiology, Pathophysiology and Experimental Endocrinology, University of Veterinary Medicine, Vienna, Austria; 4 Department of Translational Neuroscience, School for Mental Health and Neuroscience (MHeNS), Maastricht University, Maastricht, The Netherlands; 5 Department of Neurobiology, Functional Genomic Institute, CNRS /INSERM UMR 5203, University of Montpellier, 34094 Montpellier, France; 6 Center of Mental Health, Department of Psychiatry, Psychosomatics, and Psychotherapy, University Hospital of Würzburg, Würzburg, Germany; 7 Department of Neurology, University of Wuerzburg, Wuerzburg, Germany

**Keywords:** Serotonin, Tryptophan hydroxylase-2 (Tph2), Chronic stress, Gene-by-environment interaction, Anxiety, Fear, Depression, Aggression

## Abstract

**Rationale:**

While brain serotonin (5-HT) function is implicated in gene-by-environment interaction (GxE) impacting the vulnerability-resilience continuum in neuropsychiatric disorders, it remains elusive how the interplay of altered 5-HT synthesis and environmental stressors is linked to failure in emotion regulation.

**Objective:**

Here, we investigated the effect of constitutively impaired 5-HT synthesis on behavioral and neuroendocrine responses to unpredictable chronic mild stress (CMS) using a mouse model of brain 5-HT deficiency resulting from targeted inactivation of the tryptophan hydroxylase-2 (Tph2) gene.

**Results:**

Locomotor activity and anxiety- and depression-like behavior as well as conditioned fear responses were differentially affected by Tph2 genotype, sex, and CMS. Tph2 null mutants (Tph2^−/−^) displayed increased general metabolism, marginally reduced anxiety- and depression-like behavior but strikingly increased conditioned fear responses. Behavioral modifications were associated with sex-specific hypothalamic-pituitary-adrenocortical (HPA) system alterations as indicated by plasma corticosterone and fecal corticosterone metabolite concentrations. Tph2^−/−^ males displayed increased impulsivity and high aggressiveness. Tph2^−/−^ females displayed greater emotional reactivity to aversive conditions as reflected by changes in behaviors at baseline including increased freezing and decreased locomotion in novel environments. However, both Tph2^−/−^ male and female mice were resilient to CMS-induced hyperlocomotion, while CMS intensified conditioned fear responses in a GxE-dependent manner.

**Conclusions:**

Our results indicate that 5-HT mediates behavioral responses to environmental adversity by facilitating the encoding of stress effects leading to increased vulnerability for negative emotionality.

**Electronic supplementary material:**

The online version of this article (doi:10.1007/s00213-015-3879-0) contains supplementary material, which is available to authorized users.

## Introduction

Serotonin (5-hydroxytryptamine (5-HT)) is implicated in the pathophysiology of a wide spectrum of neuropsychiatric conditions, including severe depression and anxiety disorders as well as in the mechanism of antidepressant/anxiolytic pharmacotherapies. Its involvement in fundamental processes of brain development, connectivity, and plasticity identified 5-HT as a critical factor in the modulation of cognition and emotion as well as physiological and behavioral responses to stress (Lanfumey et al. 2008). While there is evidence that 5-HT moderates gene-by-environment interactions (GxE) impacting on the vulnerability-resilience continuum in neuropsychiatric disorders (for review (Lesch 2011)), the mechanisms by which environmental inputs are encoded in the brain to modify its function and behaviors are not fully understood (McEwen et al. 2012). Studies in humans report an association of functional*TPH2* variants with personality traits (Gutknecht et al. 2007) and various neuropsychiatric disorders (for review (Waider et al. 2011)). To further dissect the role of 5-HT in complex behavior and its relevance for GxE, we generated a mouse model featuring brain-specific 5-HT deficiency resulting from a targeted inactivation of neuronal tryptophan hydroxylase-2 (*Tph2*) gene (Gutknecht et al. 2008). *Tph2* null mutant (*Tph2* ^*−/−*^) mice lack the ability to synthesize 5-HT specifically in the brain (Savelieva et al. 2008; Alenina et al. 2009; Gutknecht et al. 2009, 2012). In contrast to other genetically engineered mice with brain 5-HT deficiency such as *Pet1* KO (Hendricks et al. 2003), *Lmx1b*c KO (Song et al. 2011), or *Tph2* R439H (Beaulieu et al. 2008), *Tph2* ^*−/−*^ mice completely lack brain 5-HT synthesis, while their “serotonergic-like” raphe neurons differentiate and remain electrophysiologically functional (Gutknecht et al. 2012).

Here, we investigated *Tph2* mutant mice with emphasis on the effect of central 5-HT deficiency on behavioral and neuroendocrine responses to environmental adversity. As an integral part of the neural circuitry underlying stress regulation, the 5-HT system has commonly been viewed to exert modulatory functions gender dependently (Jones and Lucki 2005). *Tph2* ^*−/−*^ mice*,* lacking brain 5-HT synthesis and *Tph2* ^*+/−*^ mice, with up to 30 % reduction of 5-HT concentrations in the raphe nuclei (Gutknecht et al. 2012; Waider et al. 2013), were subjected to unpredictable chronic mild stress (CMS (Willner 2005)) to assess the impact of deficient serotonergic neurotransmission in the brain on anxiety- and depression-like behavior, conditioned fear responses, and aggression as well as hypothalamic-pituitary-adrenal (HPA) axis reactivity and adaptation to CMS.

## Methods and materials

### Animals and experimental schedule

*Tph2* mutant mice were generated and characterized as reported previously (Gutknecht et al. 2008, 2009, 2012). Adult male and female *Tph2* ^*−/−*^, *Tph2* ^*+/−*^, and *Tph2* ^*+/+*^ mice were subjected to 3 weeks of unpredictable chronic mild stress (CMS) or assigned to a non-stressed control group (CTRL) (*n* = 8 per sex × group × genotype condition; see text S1). Mice were housed individually in a non-reversed 12/12 h light-dark cycle under controlled temperature (21 ± 1 °C) and humidity (55 ± 5 %) conditions with food and water ad libitum.

**Fig. 1:**
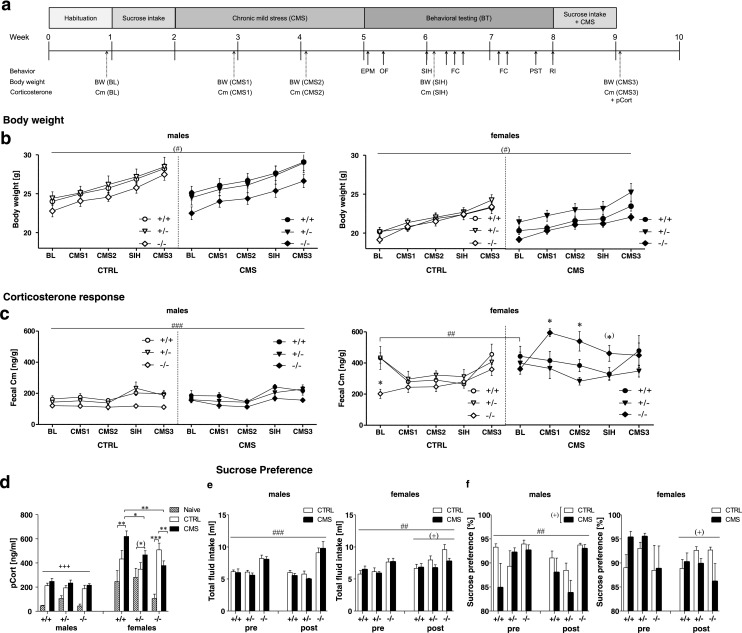
Sex-specific stress reactivity in *Tph2* ^−/−^ mice. **a** Experimental schedule. **b** Body weight of males (*left panel*) and females (*right panel*). **c** Cm values in male (*left panel*) and female (*right panel*). Control (CTRL) and chronic mild stressed (CMS) *Tph2* ^−/−^ mice were compared to *Tph2* ^+/+^ and *Tph2* ^+/−^ mice. **d** Plasma corticosterone (pCort) response in male female *Tph2* ^−/−^. Naive males: *n* = 5 *Tph2* ^+/+^ and *Tph2* ^−/−^, *n* = 4 *Tph2* ^+/−^; CTRL males: *n* = 8 *Tph2* ^+/+^, *n* = 7 *Tph2* ^+/−^ and *Tph2* ^−/−^; CMS males: *n* = 8 per genotype; naive females: *n* = 5 per genotype; CTRL females: *n* = 8 per genotype; CMS females: *n* = 7 *Tph2* ^+/+^ and *Tph2* ^−/−^, *n* = 8 *Tph2* ^+/−^. **e** Total fluid intake in males (*left panel*) and females. **f** Sucrose preference in males (*left panel*) and females (*right panel*). CTRL males: *n* = 8 *Tph2* ^+/+^, *n* = 7 *Tph2* ^+/−^ and *Tph2* ^−/−^; CMS males: *n* = 8 per genotype; CTRL females: *n* = 8 per genotype; CMS females: *n* = 8 *Tph2* ^+/+^, *n* = 8 *Tph2* ^+/−^ and *n* = 7 *Tph2* ^−/−^. *Cm*, fecal corticosterone metabolites; *pCort*, plasma corticosterone. Data are shown as means ± SEM. ^(^*^)^ *p* < 0.1, *p < 0.05, **p < 0.01, and ***p < 0.001 compared to respective controls; ^(#)^ *p* < 0.1 and ^#^ *p* < 0.05 *Tph2* ^−/−^ compared to *Tph2* ^+/+^ and *Tph2* ^+/−^; ^(+)^ *p* < 0.1, ^+^ *p* < 0.5, ^++^ *p* < 0.01, and ^+++^ *p* < 0.001 CMS vs CTRL

Sucrose preference (SP) was measured for five consecutive days before (baseline (BL)) and after behavioral testing and CMS re-exposure (BT/CMS) to assess anhedonia-like behavior. The experimental design (Fig. 1a) included elevated plus maze (EPM) and open field (OF) to investigate exploratory activity and anxiety-like behavior, stress-induced hyperthermia (SIH) to determine increases in body temperature following acute stress, a delay fear conditioning (FC) paradigm to assess associative learning and memory, a test for behavioral despair (Porsolt swim test (PST)) to analyze depression-like behavior, and the resident-intruder (RI) test to assess aggression in males (for details, see text S1).

To monitor stress responsiveness, fecal corticosterone metabolites (Cm) were analyzed according to (Touma et al. 2004) at five different time points: at BL, after 1 and 2 weeks of CMS (CMS1 and CMS2, respectively), after SIH, and after re-exposure to CMS for 1 week (CMS3). After completion of the experiments, mice were sacrificed and blood samples for plasma corticosterone (pCort) quantification were collected. An additional cohort of naive mice was used to determine baseline pCort levels.

### Statistical analysis

Data were evaluated via three-way ANOVA with sex, group, and genotype as between-subject factors followed by Bonferroni post hoc tests for significant main effects and independent *t* tests for significant interactions. Repeated measures ANOVAs (with time as within-subject factor) were performed when appropriate. Data were analyzed using IBM SPSS Statistics 21 and expressed as mean ± SEM; *p* < 0.05 was considered significant. Data were illustrated with GraphPad Prism version 5.03 for Windows, GraphPad Software, La Jolla, California USA.

## Results

### Body weight and sucrose preference

#### Body weight

Three-way ANOVA revealed a significant main effect of sex (*F*_(1,83)_ = 95.25, *p* < 0.001, Fig. 1b) and main effect of genotype (*F*_(2,83)_ = 5.09, *p* = 0.008) for body weight, indicating that females weighed less than males and *Tph2* ^*−/−*^ mice displayed lower body weights than *Tph2* ^*+/−*^ mice (*p* = 0.006). No interaction between genotype × group interaction could be detected.

#### Sucrose preference

Three-way ANOVA found a time × sex × group (*F*_(1,82)_ = 3.22, *p* = 0.076) and time × sex × genotype (*F*_(2,82)_ = 3.12, *p* = 0.050) interaction for total fluid intake; thus, data were analyzed separately for males and females (Fig. 1e). Follow-up comparisons revealed a significant genotype effect for the mean fluid intake in males (*F*_(2,41)_ = 30.36, *p <* 0.001) and females (*F*_(2,41)_ = 6.77, *p* = 0.003), showing that *Tph2* ^*−/−*^ mice of both sexes consumed more fluids than *Tph2* ^*+/−*^ and *Tph2* ^*+/+*^ mice (all *p* < 0.05). Moreover, a time × genotype interaction (*F*_(2,41)_ = 4.97, *p* = 0.012) in males indicated that total fluid intake was significantly increased in *Tph2* ^*−/−*^ mice after BT/CMS re-exposure (*p* < 0.05 compared to baseline). In females, a significant time × group interaction (*F*_(1,41)_ = 6.18, *p* = 0.017) revealed that total fluid intake did not differ between groups at baseline but tended to be increased in CTRL females compared to CMS females after BT/CMS re-exposure (*p* = 0.063).

Sucrose preference (Fig. 1f) did not significantly change over time in males. However, there was a significant genotype effect *F*_(2,41)_ = 5.59, *p* = 0.007), reflecting that *Tph2* ^*−/−*^ males exhibited increased sucrose preference as compared to *Tph2* ^*+/−*^ and *Tph2* ^*+/+*^ males (both *p* < 0.05). In addition, sucrose preference tended to be decreased in CMS males compared to CTRL males (group effect: *F*_(1,41)_ = 4.01, *p* = 0.052). In females, a time × group interaction approaching significance (*F*_(1,41)_ = 3.59, *p* = 0.065) indicated that sucrose preference was decreased in CMS females after BT/CMS re-exposure (*p* = 0.025 compared to baseline), whereas no change over time was observed in CTRL females.

A separate cohort of naive mice was used to assess daily food and water intake under baseline conditions (Fig. S2). ANOVA revealed a highly significant genotype effect for food (*F*_(2,114)_ = 31.38, *p* < 0.001) and water (*F*_(2,114)_ = 43.03, *p* < 0.001) intake (Fig. S2), reflecting that both male and female *Tph2* ^*−/−*^ mice consumed more food and water as compared to *Tph2* ^*+/−*^ and *Tph2* ^*+/+*^ mice (all *p* < 0.001). This finding is in line with increased total fluid intake in *Tph2* ^*−/−*^ mice in the sucrose preference test.

### Sex-specific HPA response to CMS

#### HPA response

Three-way ANOVA revealed a significant sex × group × genotype interaction for Cm (*F*_(2,82)_ = 3.79, *p* = 0.027). Thus, Cm data were analyzed separately for males and females. In males, ANOVA detected a significant genotype effect (*F*_(2,42)_ = 10.39, *p* < 0.001) and a tendency toward significant time × genotype interaction (*F*_(8,168)_ = 1.91, *p* = 0.062) for Cm (Fig. 1c). On average, *Tph2* ^−*/*−^ males displayed significantly lower Cm values than *Tph2* ^*+/*−^ and *Tph2* ^*+/+*^ mice (both *p* < 0.01). Moreover, 3 weeks of CMS or standard housing did not affect Cm, while SIH and further behavioral tests significantly increased Cm in *Tph2* ^*+/*−^ and *Tph2* ^*+/+*^males (all *p* < 0.05 compared to baseline Cm). In contrast, these procedures had no effect on Cm in *Tph2* ^−*/*−^ mice.

In females, ANOVA found a significant time × group (*F*_(2.96,118.35)_ = 5.03, *p* = 0.003), time × genotype (*F*_(5.92,118.35)_ = 5.26, *p* < 0.001), and group × genotype interaction (*F*_(2,40)_ = 5.07, *p* = 0.011) for Cm. Follow-up comparisons revealed that baseline Cm was significantly lower in *Tph2* ^−*/*−^ compared to *Tph2* ^*+/*−^ and *Tph2* ^*+/+*^ females (both *p* < 0.05). Moreover, Cm was significantly increased in stressed *Tph2* ^−*/*−^ females after 1 and 2 weeks of CMS as well as after SIH (all *p* < 0.05 compared to *Tph2* ^−*/*−^ controls as well as stressed *Tph2* ^*+/*−^ and*Tph2* ^*+/+*^) but not after CMS3, reflecting that *Tph2* ^−*/*−^ females were initially susceptible to CMS and BT but adapted their HPA response, while these procedures had no effect in CMS *Tph2* ^*+/*−^ and *Tph2* ^*+/+*^ females. Interestingly, the Cm response to CMS in*Tph2* ^−*/*−^ females was associated with adaptive changes in the expression of glucocorticoid receptors (GR) in the hippocampus and mineralocorticoid receptors (MR) in the frontal cortex (Supplemental Fig. S1).

For pCort, sex × group × genotype interaction approached significance (*F*_(4,103)_ = 2.43, *p* = 0.052). Follow-up comparisons revealed a significant group effect in males (*F*_(2,51)_ = 40.31, *p* < 0.001), indicating that pCort was significantly increased in CTRL and CMS males compared to naive mice (both *p* < 0.001). In females, ANOVA revealed a significant group × genotype interaction (*F*_(4,52)_ = 3.45, *p* = 0.014, Fig. 1c). CTRL *Tph2* ^−*/*−^ females exhibited significantly higher pCort levels than naive *Tph2* ^−*/*−^ mice (*p* < 0.01), whereas pCort in CTRL *Tph2* ^*+/+*^ and*Tph2* ^*+/*−^ mice did not differ from naive controls. CMS mice of all three genotypes displayed significantly elevated pCort levels as compared to naive mice of the same genotype (*Tph2* ^*+/+*^ *p* = 0.006, *Tph2* ^*+/*−^ *p* = 0.090, *Tph2* ^−*/*−^ *p* = 0.005). In addition, pCort was significantly increased in *Tph2* ^*+/+*^ females as compared to *Tph2* ^+/−^ (*p* = 0.037) and *Tph2* ^−*/*−^ mice (*p* = 0.001) within the CMS group.

#### Stress-induced hyperthermia

Three-way ANOVA revealed a significant sex × group × genotype interaction for the basal body temperature (T1) within stress induced hyperthermia (SIH) (*F*_(2,84)_ = 3.42, *p* = 0.037, Fig. 4a). In males, T1 was significantly higher in CTRL *Tph2* ^−*/*−^ (*p* = 0.011) and CMS *Tph2* ^*+/+*^ mice (*p* = 0.003) as compared to CTRL *Tph2* ^*+/+*^ mice with significant group × genotype interaction (*F*_(2,42)_ = 3.38, *p* = 0.044). By contrast, T1 did not differ between groups and genotypes in females (Fig. 4b). Furthermore, ANOVA detected a significant sex effect (*F*_(1,84)_ = 21.84, *p* < 0.001) and approached significance for a CMS effect (*F*_(1,84)_ = 3.61, *p* = 0.061) for deltaT, indicating that the increase in body temperature was higher in males compared to females and tended to be higher in CTRL vs CMS mice.

### Sex differentially affects anxiety-like behavior in *Tph2*^*−/−*^ mice

**Fig. 2:**
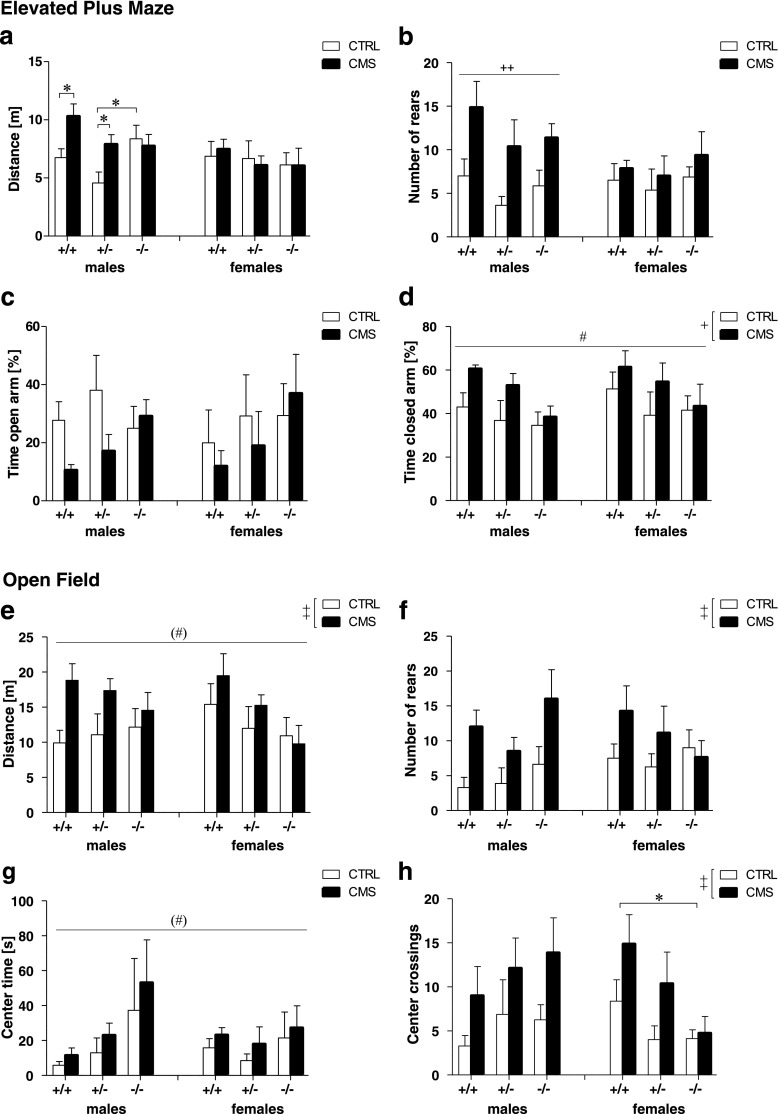
Locomotor activity and anxiety-like behavior are differentially affected by sex, stress, and *Tph2* genotype. Elevated plus maze including total distance travelled (**a**), number of rears (**b**), open arm time, (**c**) and time in closed arms (**d**) *Tph2* ^−/−^ mice compared to *Tph2* ^+/−^ and *Tph2* ^+/+^ in males (*left panel*) and females (*right panel*). Activity in the open field (OF) measured by the total distance travelled (**e**), number of rears (**f**), center time (**g**), and center crossings (**h**) in male (*upper panel*) and in female (*lower panels*) *Tph2* ^−/−^ mice. **a–d** Males: *n* = 8 per group × genotype condition except for CTRL *Tph2* ^−/−^ and CMS *Tph2* ^+/+^ (*n* = 7); females: *n* = 8 per group × genotype condition except for CMS *Tph2* ^+/+^ (*n* = 7). **e–h** Males: *n* = 8 per group × genotype condition except for CTRL *Tph2* ^+/+^ (*n* = 7); females: *n* = 8 per group × genotype condition. Data are shown as means ± SEM. ^(^*^)^ *p* < 0.1, *p < 0.05, **p < 0.01; compared to respective controls; ^(#)^ *p* < 0.1 and ^#^ *p* < 0.05 *Tph2* ^−/−^ compared to *Tph2* ^+/+^ and *Tph2* ^+/−^; ^(+)^ *p* < 0.1, ^+^ *p* < 0.5, ^++^ *p* < 0.01 CMS vs CTRL

In the EPM, three-way ANOVA revealed no significant sex × group × genotype interactions. However, sex × group interactions approached significance for the total distance traveled (*F*_(1,81)_ = 2.76, *p* = 0.101, Fig. 2a) and the number of rears (*F*_(1,81)_ = 4.01, *p* = 0.049, Fig. 2b). Follow-up comparisons revealed that CMS did not affect locomotor activity in females. However, in males, CMS mice made significantly more rears than CTRL (*F*_(1,40)_ = 14.25, *p* < 0.01). In addition, stressed *Tph2* ^*+/−*^ and *Tph2* ^*+/+*^ males traveled significantly longer distances as compared to their respective non-stressed controls, whereas stressed *Tph2* ^−/−^ males remained unaffected by CMS (group × genotype interaction: *F*_(2,40)_ = 3.01, *p* = 0.060). Moreover, CMS mice of both sexes spent significantly more time in the closed arms than CTRL (*F*_(1,81)_ = 6.06, *p* = 0.016, Fig. 2d), reflecting increased anxiety-like behavior due to CMS.

A significant genotype effect for the time spent in the closed arms (*F*_(2,81)_ = 3.63, *p* = 0.031, Fig. 2d), distance on the open arms (*F*_(2,81)_ = 3.94, *p* = 0.023, Fig. S3a) and approaching significance for % entries onto the open arms (*F*_(2,81)_ = 2.75, *p* = 0.065, Fig. S3b) argue for a reduced anxiety-like phenotype of *Tph2* ^−/−^ mice (all *p* < 0.1 compared to *Tph2* ^*+/+*^ mice). Furthermore, significant sex × genotype interactions for grooming (data not shown) and the number of defecations (Fig. S3c) indicate reduced anxiety-like behavior to be specifically prominent in *Tph2* ^−*/*−^ males, which groomed and defecated less than*Tph2* ^*+/+*^ mice (both *p* < 0.05).

In the OF, ANOVA revealed significant stress effects for the distance traveled (*F*_(1,83)_ = 7.15, *p* = 0.009, Fig. 2e), number of rears (*F*_(1,83)_ = 12.02, *p* = 0.001, Fig. 2f), center latency (*F*_(1,83)_ = 5.20, *p* = 0.025, Fig. S3e), center crossings (*F*_(1,83)_ = 10.66, *p* = 0.002, Fig. 2h), and center distance (*F*_(1,83)_ = 8.38, *p* = 0.005, Fig. S3d), indicating that CMS mice were more active and less anxious compared to CTRL mice. Moreover, a genotype effect was observed for resting time (*F*_(2,83)_ = 4.65, *p* = 0.012, data not shown) and approached significance for distance traveled (*F*_(2,83)_ = 2.48, *p* = 0.090, Fig. S3d) and time spent in the center (*F*_(2,83)_ = 2.92, *p* = 0.060, Fig. 2g), showing that *Tph2* ^−*/*−^ mice were less active and spent more time immobile than *Tph2* ^*+/+*^ mice (all *p* < 0.1). Genotype effects were particularly prominent in females, as reflected by significant sex × genotype interactions for center distance (*F*_(2,83)_ = 3.45, *p* = 0.036), center crossings (*F*_(2,83)_ = 4.00, *p* = 0.022) and defecation (*F*_(2,83)_ = 6.01, *p* = 0.004). Here, *Tph2* ^−*/*−^ females entered the center less frequently, traveled shorter distances in the center, and defecated more than *Tph2* ^*+/+*^ females (all *p* < 0.05), reflecting increased immobility and anxiety-like behavior as well as increased stress-related autonomic reactivity in the OF.

### Enhanced acquisition and retention of fear memory in *Tph2*^−*/*−^ mice

#### Acquisition of cued and contextual fear conditioning

**Fig. 3:**
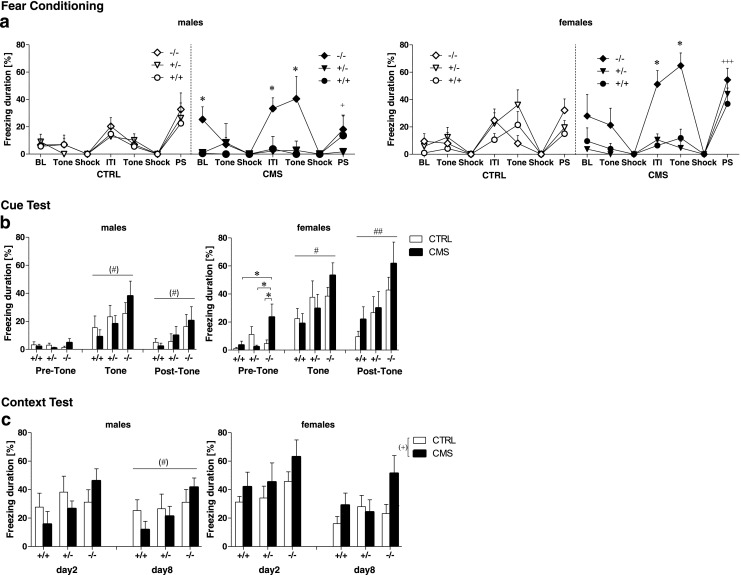
CMS differentially affects acquisition and retention of conditioned fear in *Tph2* ^−/−^ mice. **a** Freezing duration in the time course of fear conditioning training of CMS and CTRL *Tph2* mutant males (*left panel*) and females (*right panel*). Freezing was measured at baseline (BL) to the novel context, during the tone presentations, the shock, the intertrial interval (ITI), the post-shock (PS) phase, and the second tone presentation. **b** Freezing duration in the cued fear retention test of male (*left panels*) and female (*right panels*) before, during, and after tone presentation **c** Mean freezing duration in the contextual fear retention test of male (*left panels*) and female (*right panels*) in a novel context. CTRL males: *n* = 8 *Tph2* ^+/+^, *n* = 6 *Tph2* ^+/−^, *n* = 8 *Tph2* ^−/−^; CMS males: *n* = 6 *Tph2* ^+/+^, *n* = 6 *Tph2* ^+/−^, *n* = 7 *Tph2* ^−/−^; CTRL females: *n* = 8 *Tph2* ^+/+^, *n* = 8 *Tph2* ^+/−^, *n* = 7 *Tph2* ^−/−^; CMS females: *n* = 7 *Tph2* ^+/+^, *n* = 6 *Tph2* ^+/−^, *n* = 6 *Tph2* ^−/−^. Data are shown as means ± SEM. ^(^*^)^ *p* < 0.1, *p < 0.05, **p < 0.01 compared to respective controls; ^(#)^ *p* < 0.1 and ^#^ *p* < 0.05 *Tph2* ^−/−^ compared to *Tph2* ^+/+^ and *Tph2* ^+/−^; ^(+)^ *p* < 0.1; ^+^ *p* < 0.5; ^++^ *p* < 0.01, and ^+++^ *p* < 0.001 CMS vs CTRL

Repeated measure ANOVA revealed a significant phase × sex × group (*F*_(6,426)_ = 4.96, *p* < 0.001) and phase × group × genotype interaction (*F*_(12,426)_ = 5.12, *p* < 0.001) for the time spent freezing during fear conditioning (Fig. 3a). Thus, data were analyzed separately for males and females. Follow-up ANOVAs showed a significant phase × group × genotype interaction for freezing during FC in males (*F*_(12,210)_ = 1.87, *p* = 0.040) and females (*F*_(12,216)_ = 4.23, *p* < 0.001, Fig. 3a). Within a 2-min adaptation period (BL), stressed *Tph2* ^−*/*−^ males exhibited higher levels of freezing compared to stressed *Tph2* ^*+/*−^ and *Tph2* ^*+/+*^ mice (both *p* < 0.05). Although baseline freezing did not differ in females, there was a significant genotype effect for distance (*F*_(2,36)_ = 5.81, *p* = 0.007) and resting time (*F*_(2,36)_ = 6.57, *p* = 0.004, Fig. S5), reflecting that *Tph2* ^−*/*−^ females showed increased baseline immobility in the novel context during the adaptation period, similar to the behavior observed in the OF. Furthermore, stressed *Tph2* ^−*/*−^ mice of both sexes displayed significantly increased freezing during the intertrial interval (ITI) and second tone presentation (all *p* < 0.05 compared to stressed *Tph2* ^*+/*−^ and *Tph2* ^*+/+*^). Moreover, whereas CMS females froze more than controls (*p* < 0.001) after termination of the last shock (post-shock phase, PS), the opposite effect was found in males (*p* < 0.05, Fig. 3a). In addition, ANOVA revealed a significant effect of sex (*F*_(1,71)_ = 9.63, *p* = 0.003, Fig. S6) and genotype (*F*_(2,71)_ = 8.18 *p* = 0.001, Fig. S6) for the maximum velocity during the application of foot shocks, indicating that females moved faster than males and *Tph2* ^−*/*−^ mice showed increased reactivity to the shock as compared to *Tph2* ^*+/*−^ (*p* < 0.05) and *Tph2* ^*+/+*^ mice (*p* < 0.01). It has to be noticed that pain sensitivity was not significantly altered in *Tph2* ^−*/*−^ mice (Fig. S7).

#### Retention of cued fear memory

Retention of cued fear memory was tested 24 h and 7 days after conditioning. Repeated measures ANOVA revealed a significant phase × sex (*F*_(2,142)_ = 7.54, *p* = 0.001) and phase × genotype (*F*_(4,142)_ = 4.07, *p* = 0.004) interaction for freezing in the 24-h cue test (Fig. 3b) and a significant phase × sex interaction (*F*_(2,142)_ = 5.94, *p* = 0.003) for freezing in the 7-day test (data not shown). Thus, data were analyzed separately for males and females according to each phase of testing. In the 24 h test, *Tph2* ^−*/*−^ males tended to freeze more than *Tph2* ^*+/+*^ males during and after the tone presentation (both *p* < 0.1). In the 7-day test, however, enhanced freezing in *Tph2* ^−*/*−^ males was specifically observed in the tone phase (*p* < 0.1) (data not shown). In females, stressed *Tph2* ^−*/*−^ mice displayed increased freezing during the adaptation period (pre-tone) in the 24-h test (*p* < 0.05 compared to CTRL *Tph2* ^−*/*−^ as well as CMS *Tph2* ^*+/+*^ and CMS *Tph2* ^*+/*−^ females). Moreover, *Tph2* ^−*/*−^ females of both groups froze significantly more than *Tph2* ^*+/+*^ mice during tone presentation and the post-tone phase (both *p* < 0.05). Similar results were observed for the 7-day test.

#### Retention of contextual fear memory

Retention of contextual fear memory was tested 48 h and 8 days after conditioning (Fig. 3c). Three-way ANOVA revealed a significant sex effect (*F*_(1,71)_ = 5.72, *p* = 0.019) and genotype effect (*F*_(2,71)_ = 3.75, *p* = 0.028) for freezing during the 48-h context test and a group × genotype (*F*_(2,71)_ = 2.47, *p* = 0.092) and sex × group interaction (*F*_(1,71)_ = 2.66, *p* = 0.107) approaching significance for freezing in the 8-day context test; thus, males and females were analyzed separately for each testing phase. In males, no significant differences were observed for freezing in the 48-h test. In the 8-day test, however, *Tph2* ^−*/*−^ males tended to freeze more than *Tph2* ^*+/+*^ males (*p* < 0.1). In females, a stress effect approached significance for freezing in the 48-h and 8 day test (both *p* < 0.1), indicating that CMS females tended to freeze more than CTRL mice. In general, CMS *Tph2* ^−*/*−^ mice froze more in the context test compared to *Tph2* ^−*/*−^ CTRL (*p* < 0.05) and compared to stressed *Tph2* ^*+/*−^ and *Tph2* ^*+/+*^ (*p* < 0.05) especially at day 8.

### Altered depression-like behavior in *Tph2*^−*/*−^ mice

**Fig. 4:**
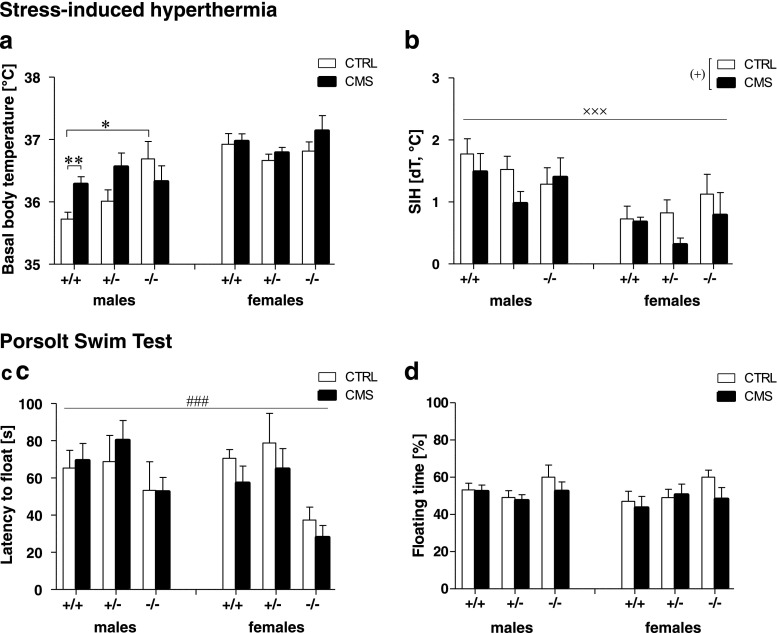
Sex, stress, and *Tph2* genotype differentially affect temperature control and depression-like responses. **a**, **b** Stress-induced hyperthermia of chronic mild stressed (CMS) *Tph2* ^−/−^ mice compared to*T ph2* ^+/+^ and *Tph2* ^+/−^ mice and their respective controls (CTRL) with basal body temperature for males (*left panel*) and females (*right panel*) (**a**) and the increase in body temperature (dT) (**b**). Latency to float (**c**) and total floating time (**d**) in the Porsolt swim test in male (*left panel*) and female *Tph2* ^−/−^ (*right panel*) mice; *n* = 8 per sex × group × genotype condition except for female CMS *Tph2* ^+/+^ and *Tph2* ^−/−^ (*n* = 7). Data are shown as means ± SEM. ^(^*^)^ *p* < 0.1, *p < 0.05, and **p < 0.01 compared to respective controls; ^(#)^ *p* < 0.1 and ^###^ *p* < 0.001 *Tph2* ^−/−^ compared to *Tph2* ^+/+^ and *Tph2* ^+/−^; ^(+)^ *p* < 0.1 CMS vs CTRL; ^xxx^ *p* < 0.001 males vs females

In the PST (Fig. 4c–d), three-way ANOVA revealed no interactions of sex with stress and genotype. However, a highly significant genotype effect for the latency to float (*F*_(2,82)_ = 8.62, *p* < 0.001, Fig. 4c) and a tendency toward a genotype effect for the time spent swimming (*F*_(2,82)_ = 2.69, *p =* 0.074, Fig. S4) was detected. *Tph2* ^−*/*−^ mice gave up struggling earlier than *Tph2* ^*+/*−^ and *Tph2* ^*+/+*^ (both *p* < 0.05) and tended to swim less than *Tph2* ^*+/+*^ mice (*p* = 0.1). However, total floating duration did not significantly differ between genotypes (*F*_(2,82)_ = 2.18, *p* = 0.119) (Fig. 4d).

### Increased aggression in *Tph2*^−*/*−^ males

**Fig. 5:**
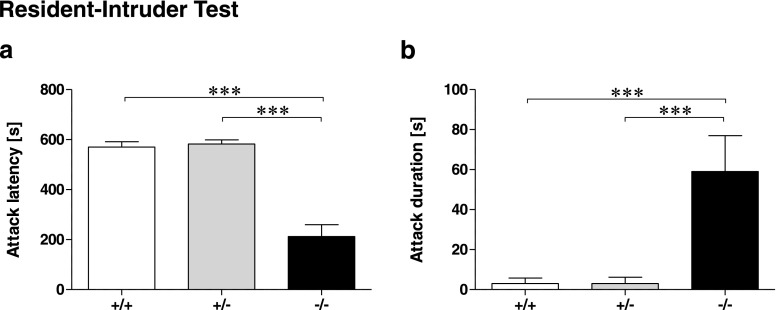
Aggression in male *Tph2* ^−/−^ mice. **a** Latency to attack of *Tph2* ^−/−^ mice onto the intruder mouse and (**b**) cumulative attack durations of *Tph2* ^−/−^ mice compared to *Tph2* ^+/+^ and *Tph2* ^+/−^ mice. *n* = 6 per group × genotype condition. Data are shown as mean ± SEM. ***p < 0.001

ANOVA revealed a significant genotype effect for the latency to attack (*F*_(2,30)_ = 44.43, *p* < 0.001) and total attack duration (*F*_(2,30)_ = 10.29, *p* < 0.001) in the RI test (Fig. 5). *Tph2* ^−*/*−^ males showed reduced attack latencies and increased cumulative attack durations compared to *Tph2* ^*+/*−^ and *Tph2* ^*+/+*^ males (all *p* < 0.001).

## Discussion

### 5-HT and sex-dependent HPA response to CMS

Sex-specific functions of 5-HT have previously been reported (Bethea et al. 1998, 2009; Rubinow et al. 1998; Goel and Bale 2010; Hall and Steiner 2013). Our results show that life-spanning inactivation of brain Tph2 synthesis differentially impacts behavior in both male and female *Tph2* ^−*/*−^ mice and their response to CMS. Sex-specific stress responses due to CMS were described in both rodents and humans, suggesting an interaction between the HPA and hypothalamic-pituitary-gonadal (HPG) systems (Goel and Bale 2010) and references therein. Previous reports of pCort levels following CMS in rodents yielded conflicting results with either reduced (Murison and Hansen 2001), unchanged (Azpiroz et al. 1999), or increased concentrations after 3 weeks (Bielajew et al. 2002) or after 1 and 2 weeks but with return to baseline after 3–6 weeks (Silberman et al. 2002). In patients with severe depressive disorders, sex-related dysregulation of the HPA system with higher circadian cortisol secretion and impaired negative feedback regulation was generally but not consistently reported (Posener et al. 2000; Gold and Chrousos 2002; Stewart et al. 2005; Lanzenberger et al. 2010; Parihar et al. 2011; Wardenaar et al. 2011); for review (Pariante and Lightman 2008). In this study,

*Tph2* ^−*/*−^ females showed a strong rise in Cm levels during CMS while *Tph2* ^−*/*−^ males remained insensitive, suggesting increased stress vulnerability and activation of the HPA system in *Tph2* ^−*/*−^ females. Furthermore, stress-naive *Tph2* ^−*/*−^ females displayed low Cm levels, suggesting hypoactivity of the HPA system under basal conditions due to the lack of 5-HT. Thus, CMS produced an overactivation of the HPA axis, which is able to adapt after multiple CMS exposures. Interestingly, 5-HT transporter (5-Htt) deficient mice also showed stress-induced alterations in the adrenocortical stress response as measured by Cm excretion levels (Jansen et al. 2010). As 5-HT was shown to alter HPA system activity and brain corticosteroid receptor expression (Goel and Bale 2010; Heydendael and Jacobson 2010), an increased HPA activation in *Tph2* ^−*/*−^ females is likely due to a cumulative effect of 5-HT deficiency and gonadal steroids, supporting the notion of hormone-dependent adaptive mechanisms to stress in females vs males. In line with this, corticotropin-releasing factor (CRF) injections increased cFos expression in males but decreased cFos in females in the dorsal raphe (Howerton et al. 2014).

### Depression-like behavior in *Tph2*^−*/*−^ females

Discrepant results were reported for depression-like behavior in *Tph2* ^−*/*−^ mice (Savelieva et al. 2008; Mosienko et al. 2012) as well as in Tph2 R439H mutant mice (Beaulieu et al. 2008). Here, we report a reduced latency to float and a trend to lower swimming time in *Tph2* ^−*/*−^ mice in the PST, suggesting a tendency to behavioral despair. However, the total floating duration was not altered in *Tph2* ^−*/*−^ mice. Although consistently supported in a review by Willner (Willner 2005), we did not detect an effect of CMS on floating time in the PST, which was conducted at the end of BT. However, the preference for sucrose decreased in CMS females after BT/CMS re-exposure, thus arguing for an anhedonia-like effect of CMS in females independent of genotype. In contrast to females, sucrose preference was increased in *Tph2* ^−*/*−^ males, whereas CMS had no effect. Interestingly, *Tph2* ^−*/*−^ mice of both sexes showed a general increase in total liquid and food intake but remained thinner compared to controls. This can be explained by an effect of lifelong Tph2 deficiency on metabolism, with *Tph2* ^−/−^ mice displaying an increased energy metabolism (Yadav et al. 2009). Because a high metabolism and an increased level of activity in the open field were associated with a long latency to float in the PST representing active stress coping behavior (Campbell et al. 2003), *Tph2* ^−*/*−^ mice might display a shorter latency to float due to their altered metabolism rather than a depressive-like phenotype (Mosienko et al. 2014), which manifests itself differentially in males and females.

### 5-HT deficiency prevents males from stress-induced hyperlocomotion

Previous studies provided inconsistent evidence for altered anxiety-like behavior in *Tph2* ^−*/*−^ mice (Angoa-Perez et al. 2012; Mosienko et al. 2012). Our results demonstrate that anxiety-like behavior in *Tph2* mice is moderated by sex, stress, and genotype. Interestingly, *Tph2* ^−/−^ mice showed reduced anxiety-like behavior in the EPM indicated by reduced time spent in the closed arms. Thus, 5-HT seems to tonically reduce risk assessment behavior, defined as frequent approach and withdrawal movements providing information of potential dangerous situations leading to either further defensiveness or a return to non-defensive behaviors (Blanchard and Blanchard 1989). Based upon lifelong 5-HT depletion in the brain, *Tph2* ^−/−^ males do not respond to CMS with increased locomotor activity like *Tph2* ^+/+^ and *Tph2* ^+/−^ males. Thus, CMS may induce hyperlocomotion through 5-HT-dependent mechanisms (Strekalova et al. 2005; Hale et al. 2008; Couch et al. 2013), potentially involving different subgroups of 5-HT neurons targeting different brain regions involved in locomotor control (Hale et al. 2012). In line with present and other studies, deficiency of 5-HT in males may shift defensive avoidance behavior toward active avoidance (Angoa-Perez et al. 2012; Mosienko et al. 2012). While male *Tph2* ^−*/*−^ showed reduced anxiety in the EPM and OF, *Tph2* ^−*/*−^ females exhibited increased anxiety, as reflected by novelty-induced locomotor suppression and increased stress-related autonomic reactivity in the OF but reduced anxiety in the EPM without an increase in locomotion. Thus, 5-HT-dependent mechanisms seem to act in females via tonic suppression of anxiety in escapable contexts like the EPM, while in inescapable contexts, 5-HT serves to suppress an increased stress response and passive coping behaviors, which is supported by increased baseline immobility of *Tph2* ^−*/*−^ females during the adaptation period in fear conditioning.

In contrast to unconditioned anxiety-like behavior in the EPM, fear conditioning provides an inescapable aversive physical stimulus evoking an adaptive freezing response to the novel context (Homberg 2012) similar to the OF. Furthermore *Tph2* ^−*/*−^ mice showed increased shock reactivity independent of CMS, which indicates that 5-HT is implicated in the suppression of flight responses (Johnson et al. 2004; Spannuth et al. 2011; Gutknecht et al. 2012), as thermal and tactile pain sensitivity was not changed in *Tph2* ^−*/*−^ mice.

Increased cue fear learning in male and female *Tph2* ^−*/*−^ mice is in line with findings in other models of 5-HT deficiency (Dai et al. 2008; Kiyasova et al. 2011) and argue for specific effects of 5-HT neuron subgroups, which balance CMS-evoked changes in limbic brain areas involved in cued conditioning (Mongeau et al. 1997; Sullivan et al. 2004; Pape and Pare 2010; Hale et al. 2012).

However, long-term contextual fear conditioning was influenced by the lack of 5-HT in stressed mice, arguing for 5-HT as important factor in the inhibition of contextual long-term memory formation after CMS. Furthermore, the correlation of Cm with an increase in MR expression in the frontal cortex of *Tph2* ^−*/*−^ females after 1 week of CMS indicates a reciprocal modulation between the HPA axis and the 5-HT system mediated by differential expression of GR and MR receptors of males and females in the frontal cortex and the hippocampus. This, in turn, may modulate *Tph2* and 5-HT receptor expression (Lanfumey et al. 2008; Nexon et al. 2011; Gutknecht et al. 2012), which are involved in contextual memory formation and points toward a similar effect of CMS and inescapable stress on the prefrontal cortex, which may inhibit 5-HT mediated stress-related adverse effects (Amat et al. 2005; Baratta et al. 2009).

Here, we used unpredictable CMS in combination with a behavioral testing battery. From a clinical perspective, it appears counterintuitive that 5-HT deficiency simultaneously results in anxiolytic effects and in depression-like behavior. Nevertheless, mirror models like the 5-HT_1A_ KO mouse also display increased anxiety-like and reduced depressive-like behavior (Heisler et al. 1998). In patients, depression is frequently accompanied by anxiety symptoms, also being responsive to drugs targeting 5-HT neurotransmission. Lemogne and associates recently suggested that cognitive appraisal modulates brain responses to emotional stimuli and can counteract genetic and environmental susceptibility factors in humans (Lemogne et al. 2011). Furthermore, CMS may induce diverse compensating effects based on the lifelong inactivation of brain 5-HT synthesis involving activity-induced adult neurogenesis (Parihar et al. 2011; Klempin et al. 2013). Thus, a simplified, more accessible and controllable model such as the *Tph2* ^−*/*−^ mouse may help deciphering basic mechanisms and neuronal circuits involved in the different roles of 5-HT likely to be operant in humans as well. Taken together, our findings suggest that 5-HT depletion throughout life blocks the CMS effect on locomotion and anxiety in males and in females. Thus, 5-HT seems to mediate the locomotor stress effects and that it is of clinical relevance to understand the neural network and adaptive mechanisms between sexes, which allow anxiety, fear, and depressive symptoms to be ameliorated by mild stressors of an enriched environment like CMS.

### Impulsive and hyperaggressive behavior in *Tph2*^−*/*−^ males

In the RI paradigm, *Tph2* ^−/−^ males exhibited highly increased aggressive behavior, particularly increased impulsivity reflected by decreased latency to the first attack and increased duration of fighting, which was further aggravated by CMS. Aggression-like behavior of a male resident toward male intruders is an ethologically determined response to territorial threats. Overwhelming evidence links 5-HT to impulsive and aggressive behavior as the primary determinant of aggression control (Lesch and Merschdorf 2000). Several regions of the frontal and cingulate cortices, amygdala, septum, hypothalamus, and periaqueductal gray matter are among the best documented to be involved in the neural circuitry of aggression. Serotonergic fibers extensively project to each of these regions, and it is well established that both aggressiveness and increased impulsivity are associated with brain 5-HT deficiency. The impulsive and hyperaggressive behavior of *Tph2* ^−/−^ mice resembles the increased defensiveness reported for *Pet1* KO (Hendricks et al. 2003) and *Tph2* R439H mutants (Beaulieu et al. 2008). Acute treatment with 5-HT_1A_ and 5-HT_1B_ receptors agonists (or 5-HT_2A/2C_ antagonists) via their inhibitory action on neurotransmission (presynaptically or postsynaptically) was reported to reduce aggressive behavior, and it was suggested that low 5-HT levels in the brain are associated with maladaptive forms of excessive violence rather than with natural defensiveness (Miczek et al. 2007; de Boer et al. 2009). Furthermore, chronic reduction in serotonin neuron firing through overexpression of Htr1a in adulthood, but not during development, was sufficient to increase aggression (Audero et al. 2013). Since 5-HT deficiency is likely to result in impaired inhibition of engagement and sustainment of aggressive behavior, it may explain that *Tph2* ^−/−^ mice display exaggerated aggressive behavior as a consequence of failing 5-HT-mediated inhibitory control, thus rendering these mice inert to acquire social abilities.

In contrast to *Tph2* ^−*/*−^ mice, no effects of sex and CMS on behavior were detected in *Tph2* ^*+/*−^ mice, which are characterized by up to 30 % reduction in 5-HT in the raphe but unaltered concentrations of 5-HT in target regions of 5-HT neurons (Gutknecht et al. 2012). This provides evidence for a specific cellular compensatory mechanism during development in *Tph2* deficient mice, which affects GABAergic networks via upregulation of 5-HT1A and 5-HT2 receptors (Jorgensen et al. 2013; Waider et al. 2013).

In conclusion, converging lines of evidence suggest that the balance of the 5-HT system plays a critical role in synaptic plasticity of a multitude of neuronal networks, thus setting the stage for expression of complex traits and their associated behaviors throughout development and adult life depending on the individual 5-HT metabolism (Lesch and Waider 2012). Moreover, variation in genes moderating 5-HT system function, in conjunction with other rare and common variants of the genetic background and with inadequate adaptive responses to environmental stressors, is also likely to contribute to negative emotionality and aggression-related behavior emerging from compromised brain development and from highly efficient neuroadaptive processes across the life cycle.

## Electronic supplementary material

Below is the link to the electronic supplementary material.

ESM 1 (DOCX 1352 kb)
