# Supplementary material for: Interaction of brain 5-HT synthesis deficiency, chronic stress and sex differentially impact emotional behavior in Tph2 knockout mice
Source: Psychopharmacology (Berl). 2015 Feb 27;232(14):2429–41. doi: 10.1007/s00213-015-3879-0 (PMC4480945; doi:10.1007/s00213-015-3879-0)
Supplement: Supplementary file 1 — (DOCX 1352 kb) [file 213_2015_3879_MOESM1_ESM.docx]

**Supplemental Information**

In order of appearance in the manuscript

**Text S1: Supplemental Methods and Materials**

**Integrated study in the same animals**

**General information**

All animal protocols were performed after they had been approved by the review board of the Government of Lower Franconia and the University of Würzburg and according to the European Community guidelines for animal care (DL 116/92, application of the European Communities Council Directive 86/609/EEC).

**Sucrose consumption and chronic mild stress (CMS)**

24 male and 24 female *Tph2^+/+^, Tph2*^+/-^ and *Tph2*^-/-^ mice (*n*=8 per sex and genotype) were chronically exposed to unpredictable mild stressors for 21 consecutive days. The CMS procedure followed a fixed weekly schedule (described in the table below) and consisted of a variety of commonly used stressors, including one period of overnight illumination (continuous light for 36h), one period of food deprivation for 15h, one period of water deprivation for 15h, three periods of tilted cage (30°) for 3h, three periods of exposure to an empty bottle for 3h, and three periods of restraint stress (confinement to a restricted space of 8x8x11cm within the home cage) for 1h. An additional cohort of 24 male and 24 female *Tph2^+/+^, Tph2*^+/-^ and *Tph2*^-/-^ mice (*n*=8 per sex and genotype) served as control group for subsequent behavioral experiments. During CMS, control mice remained undisturbed in the home cage except for standard handling procedures.

Baseline sucrose and water consumption was measured for five consecutive days immediately before to the onset of CMS. Mice were given a free choice between two bottles, one with 2.5% sucrose solution and the other with tap water. The consumption of water, sucrose solution and total intake of liquids was measured simultaneously in control and CMS groups by weighing the bottles every 24h. Sucrose consumption was calculated as mean sucrose intake per day over five days. After behavioral testing, mice of the stress groups were re-exposed to CMS for five consecutive days and sucrose consumption was measured again at the same time.

**Chronic mild stress time schedule**

|  | Time | Monday | Tuesday | Wednesday | Thursday | Friday | Saturday | Sunday |  |
| --- | --- | --- | --- | --- | --- | --- | --- | --- | --- |
|  | 06:00 |  |  |  |  |  |  |  |  |
|  | 07:00 |  |  |  |  |  |  |  |  |
|  | 08:00 |  |  |  |  |  |  |  |  |
|  | 09:00 |  |  | Restraint | Tilted cage | Empty bottle | Tilted cage | Restraint |  |
|  | 10:00 |  |  |  |  |  |  |  |  |
|  | 11:00 |  |  |  |  |  |  |  |  |
|  | 12:00 |  |  |  |  |  |  |  |  |
|  | 13:00 |  |  |  |  |  |  |  |  |
|  | 14:00 |  |  |  |  |  |  |  |  |
|  | 15:00 |  | Tilted cage | Empty bottle |  |  |  | Empty bottle |  |
|  | 16:00 |  |  |  |  |  |  |  |  |
|  | 17:00 |  |  |  |  | Restraint |  |  |  |
|  | Overnight | Water |  |  | Food |  | Overnight |  |  |
|  |  | deprivation |  |  | deprivation |  | illumination |  |  |
|  |  |  |  |  |  |  |  |  |  |

**Elevated plus maze (EPM)**

The plus-shaped maze was made of grey Perspex (TSE Systems Inc., Bad Homburg, Germany) and comprised two opposing open arms (30x5x0.25cm) and two opposing closed arms (30x5x15cm) extending from a central platform (5x5cm). The device was elevated to a height of 60cm above the floor. Illumination intensity was 200lux on the open arms, 120lux on the central platform and 40lux on the closed arms. Mice were placed in the center, facing one of the open arms, and allowed to freely explore the maze for 5min. The number of arm entries, the time spent in the open and closed arms and the total distance traveled were recorded using an automated video tracking system (VideoMot2, TSE Systems Inc., Bad Homburg, Germany). In addition, the number of vertical rears and grooming bouts as well as defecation (fecal boli and urine) were scored.

**Open field (OF)**

The open field consisted of a square-shaped box (50x50x40cm) made of Perspex XT, a black opaque material semi-permeable for infrared (IR) light (TSE Systems Inc., Bad Homburg, Germany). The apparatus was brightly illuminated by IR LED’s from below. Activity monitoring was conducted using an IR sensitive CCD camera (GKB Security Corporation, Taichung, Taiwan) along with the computerized video tracking software VideoMot2 (TSE Systems Inc., Bad Homburg, Germany). The OF arena was divided into a small center zone (25x25cm) and the surrounding periphery. Illumination at floor level was 200lux. Mice were placed close to the wall and allowed to freely explore the arena for 5min. Variables measured included the total distance traveled, the time spent in the center and periphery, the number of center crossings, the number of vertical rears and grooming bouts as well as defecation (fecal boli and urine).

**Stress-induced hyperthermia (SIH)**

In the first trial, the basal body temperature (T1) of the mice was measured by inserting a digital microprobe thermometer (BAT-12; **Physitemp Instruments, Inc.**) into the rectum until the readout was stable (approx.10-20s). Measuring T1 is considered an acute stressor causing a rise in core body temperature within a few minutes. After an intertrial-interval of 10min, the rectal temperature of the mice was measured again (T2) and the SIH response was calculated as the difference between T2 and T1: SIH=deltaT=T2-T1.

**Fear Conditioning**

Delay fear conditioning was conducted using an automated fear conditioning system (TSE Systems Inc., Bad Homburg, Germany). The testing chamber was consistently illuminated with a light intensity of 100lux. During fear conditioning training (day 0), mice were placed into the conditioning chamber and allowed to explore the novel environment for 2min (baseline) before an 80dB, 10kHz sine tone (auditory cue, conditioned stimulus, CS) was presented for 20s. During the last 2s of the tone, a 0.8mA scrambled foot shock (unconditioned stimulus, US) was administered. An 80s intertrial interval preceded a second identical CS-US trial. Mice remained in the chamber for further 60s (post-shock phase) before they were returned to their home cage. Recall of cue fear memory testing was performed at day 1 and day 7 after conditioning. Mice were tested in the same chamber, but in an altered context: the grid floor was covered by a grey PVC plate and woodchip bedding; the walls were surrounded by posters to diminish the field of vision and a novel olfactory cue was provided using 3% acetic acid solution for cleaning instead of 70% alcohol. Mice were placed into the chamber and allowed to explore the ‘novel’ environment for 2min (pre-tone phase). Subsequently, the auditory cue was presented for 150s. After termination of the tone, mice remained in the chamber for 30s (post-tone phase) before they were returned to their home cage. Contextual fear testing was conducted at day 2 and day 8 after conditioning. Mice were placed into the original conditioning context and allowed to explore the environment without presentation of the tone for 5min. Mouse behaviors, including activity, resting, freezing and rearing, were automatically monitored via infrared light beams. Freezing was defined as complete immobility for >3s.

**Fear conditioning protocol**

|  |  |  |  |  |  |  |  |  |  |  |
| --- | --- | --- | --- | --- | --- | --- | --- | --- | --- | --- |
|  |  |  |  |  |  |  |  |  |  |  |
| Fear conditioning | | |  | Auditory cue test | | |  | Context test | | |
| (day 0) | | |  | (day 1 and day 7) | | |  | (day 2 and day 8) | | |
|  |  |  |  |  |  |  |  |  |  |  |
|  |  |  |  |  |  |  |  |  |  |  |
| 120s |  | Baseline |  | 120s |  | Pre-tone |  | 300s |  | Context |
| 18s |  | Tone |  | 150s |  | Tone |  | **300s** |  |  |
| 2s |  | Tone + Shock |  | 30s |  | Post-tone |  |  |  |  |
| 80s |  | Intertrial interval |  | **300s** |  |  |  |  |  |  |
| 18s |  | Tone |  |  |  |  |  |  |  |  |
| 2s |  | Tone + Shock |  |  |  |  |  |  |  |  |
| 60s |  | Post-shock |  |  |  |  |  |  |  |  |
| **300s** |  |  |  |  |  |  |  |  |  |  |
|  |  |  |  |  |  |  |  |  |  |  |

**Porsolt swim test (PST)**

Mice were introduced to a transparent cylinder (height 20cm, diameter 15cm) filled with water (25°C, height 12cm) and parameters of floating behavior, including the latency to float and cumulative floating duration, were scored for 5min. Floating was defined as complete immobility except for minimal movements necessary to keep the head above water.

**Resident-intruder test**

A stimulus male mouse (intruder) was introduced into the home cage of the resident male mouse and the latency to the first attack, the cumulative duration and the total number of attacks initiated by the resident mouse were recorded for 10min.

**Fecal corticosterone metabolites (Cm)**

To avoid additional stress, the collection of fecal boli was performed during regular cage change. Mice were placed into a novel cage and all fecal boli produced within 24h were collected, dried for 2h at 80°C and powdered with mortar and pestle. Aliquots of 50mg were mixed with 1ml 80% methanol, vortexed for 3x30s and centrifuged for 10min at 2500g. The supernatants were further analyzed with a 5α-pregnane-3β,11β,21-triol-20-one enzyme immunoassay (EIA) as previously described (7). This group-specific EIA detects steroids with a 5α-3β,11β-diol structure and has been successfully validated for measuring adrenocortical activity in mice (45). Intra- and inter-assay coefficients of variation were 9.1% and 11.1%, respectively and sensitivity of this EIA was 1 ng/0.05 g feces.

**Plasma corticosterone (pCORT)**

Blood was collected by intracardial puncture under deep anesthesia. Plasma was separated by centrifugation (4°C, 20min) and pCORT was measured by standard radioimmunoassay (RIA) using the ImmuChem^TM^ Double Antibody Corticosterone ^125^I RIA Kit for mice (MP Biomedicals). The assay was performed according to the instructions given by the manufacturer.

**Quantitative real-time PCR (qRT-PCR) for mineralocorticoid (MR) and glucocorticoid receptors (GR)**

After completion of behavioral testing, mouse brains were collected, immediately frozen through immersion in precooled 2-methyl butane on dry ice and stored at -80°C until use. Brain regions were rapidly dissected on a cold plate and immediately re-frozen on dry ice. Tissues were homogenized in peqGold RNA pure buffer (PeqLab) by shaking with Tissuelyser (Qiagen). Total RNA was extracted using RNeasy Kit (Qiagen) in conjunction with DNase treatment. Quality, purity and concentration of the samples were assessed by photometry and electrophoresis. cDNA was synthesized from 1 µg total RNA using iScript™ cDNA Synthesis Kit (Bio-Rad). Quantitative real-time PCR (qRT-PCR) was performed using the QuantiFast SYBR green mix (Qiagen) in 384 wells plates placed in the CFX384^TM^ real-time PCR system from Biorad. Analysis of the raw data was performed with LinRegPCR (1). For the quantification of MR and GR gene expression, QuantiTect primers (Qiagen) for their respective genes, Nr3c2 and Nr3c1, were used. All qRT-PCRs were performed in triplicates and normalized against the 2 most stable reference genes out of 4 (*Rn18S*, *Actb*, *Gapdh*, *Ubc*) chosen by using the geNorm algorithm (2). Data represent the normalized relative expression levels.

**Supplemental Results**

*r*=0.12, *p*=0.64

*r*=0.6, *p*=0.023

**c**

**b**

**a**


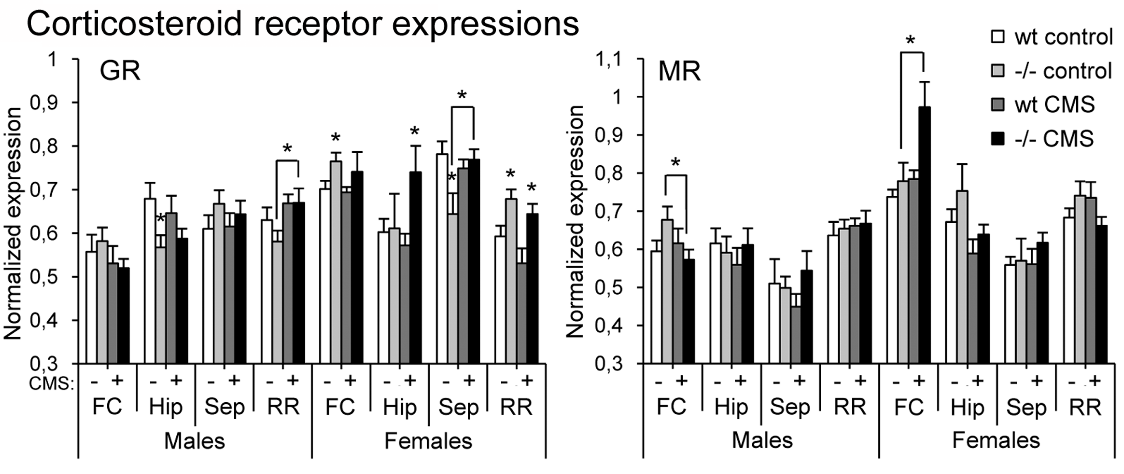

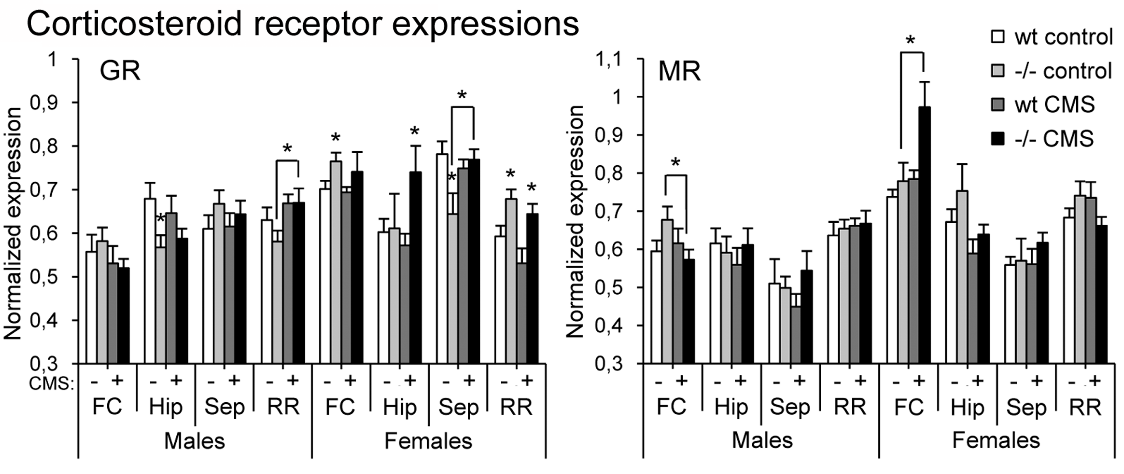

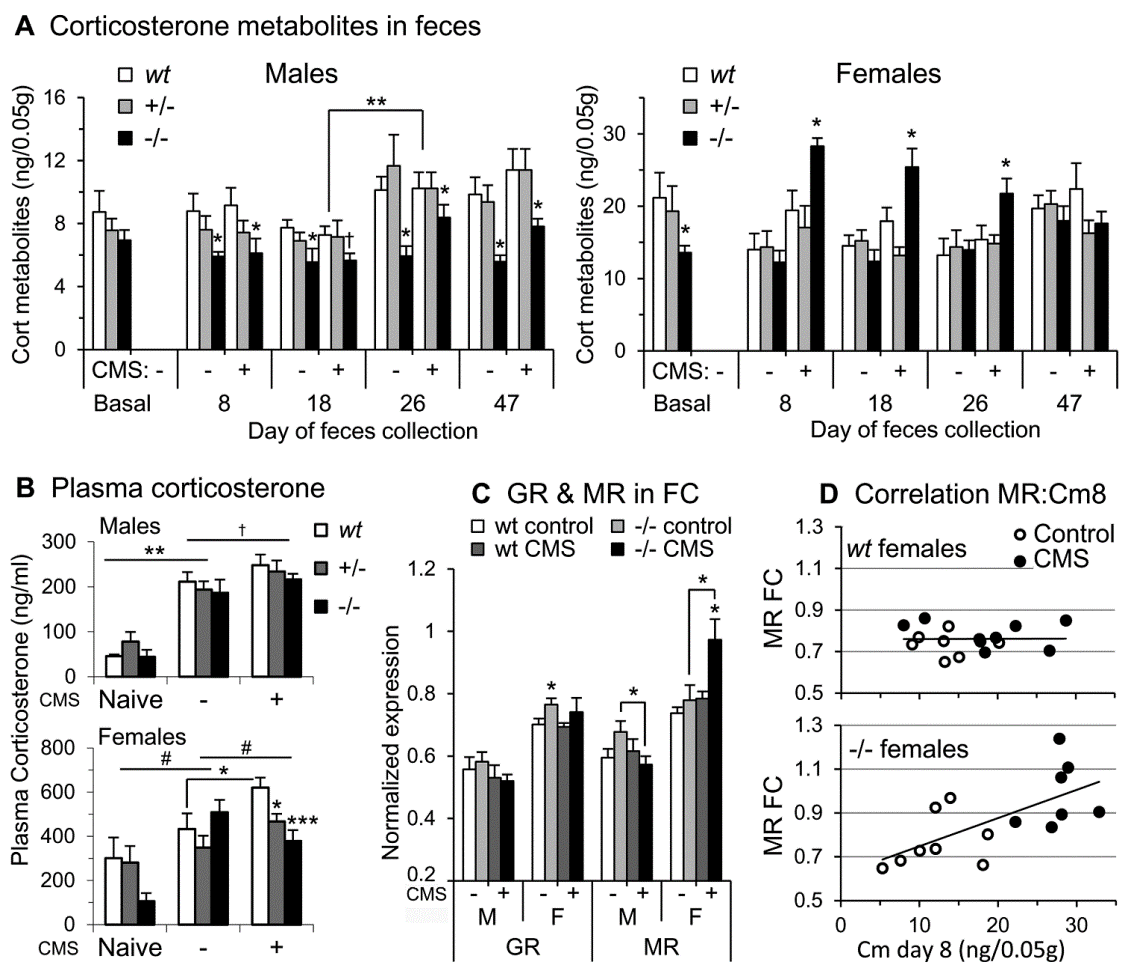


**Fig. S1: Sex-specific changes in the expression of glucocorticoid and mineralocorticoid receptors in response to CMS. (a)** Glucocorticoid receptor (GR) and **(b)** mineralocorticoid receptor (MR) expression in the frontal cortex (FC), hippocampus (Hip), septum (Sep) and rostral raphe (RR). **(c)** Correlation between MR expression in the FC and fecal corticosterone metabolite concentrations (Cm) after exposure to CMS for 8 days. **(a)** In males, GR expression was significantly lower in the Hip of non-stressed *Tph2*^-/-^ mice compared to *Tph2*^+/+^ controls. By contrast, GR expression was significantly upregulated in the RR of stressed *Tph2*^-/-^ mice compared to their non-stressed *Tph2*^-/-^. In female *Tph2*^-/-^ controls, significantly higher GR expression was found in the FC and RR, whereas lower GR levels were found in the Sep (all compared to *Tph2*^+/+^ controls). However, reduced GR expression in the Sep of *Tph2*^-/-^ females was normalized after CMS. Moreover, CMS significantly increased GR expression in the Hip and RR of *Tph2*^-/-^ females (compared to stressed *Tph2*^+/+^) and in the Sep (compared to *Tph2*^-/-^ controls). **(b)** Whereas male *Tph2*^-/-^ controls displayed significantly higher MR expression levels than stressed *Tph2*^-/-^ males in the FC, the opposite effect was found in *Tph2*^-/-^ females, where CMS significantly increased MR expression in the FC. **(c)** MR expression in the FC was positively correlated with fecal Cm concentrations after exposure to CMS for 8 days in female *Tph2*^-/-^mice (lower panel), whereas no relation between MR expression and fecal Cm was found in *Tph2*^+/+^ mice (upper panel). *n*=8 except for CMS *Tph2*^-/-^ females (*n*=7). Data are shown as means±SEM. **p*<0.05.


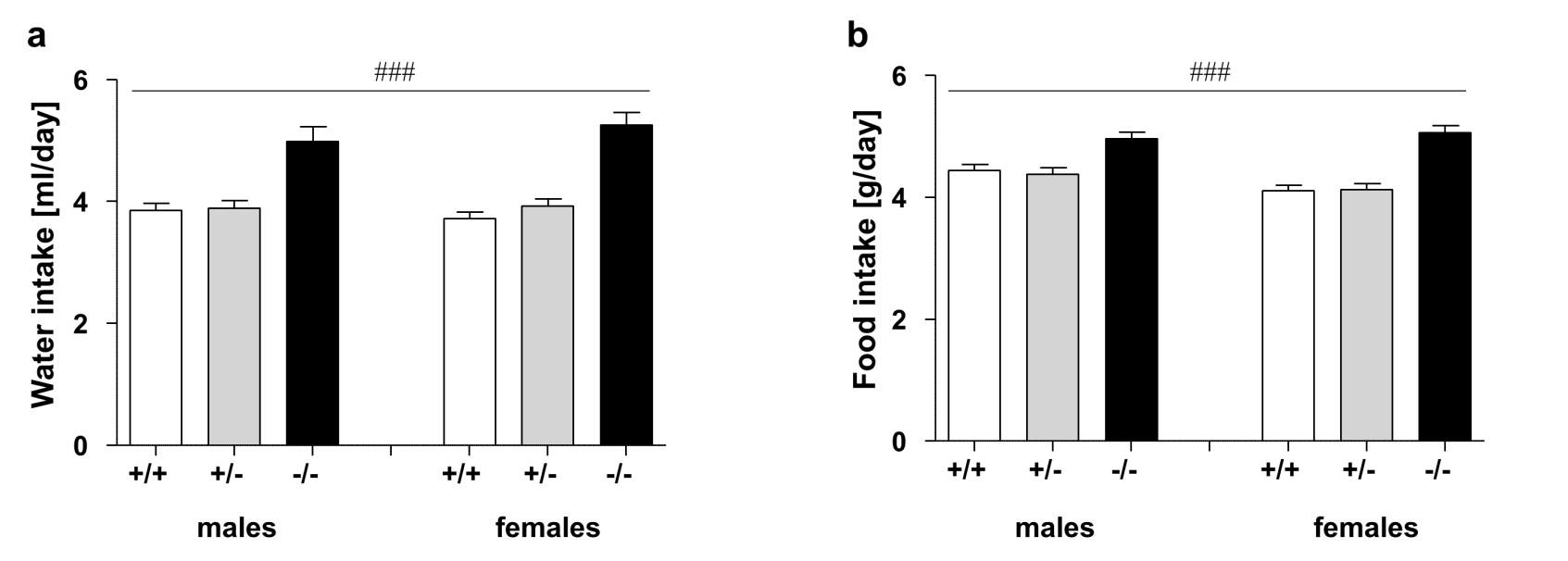


**Fig. S2: Metabolism is increased in Tph2^-/-^ mice independent of sex**

**(a)** total water intake and (b) total food intake of *Tph2^-/-^* compared to *Tph2^+/-^* and *Tph2^+/+^* males and females. males: *n*=17/genotype*;* females: *n*=23/genotype.

Data are shown as means ± SEM. ^###^p<0.001 Tph2^-/-^ compared to Tph2^+/-^ and Tph2^+/+^.


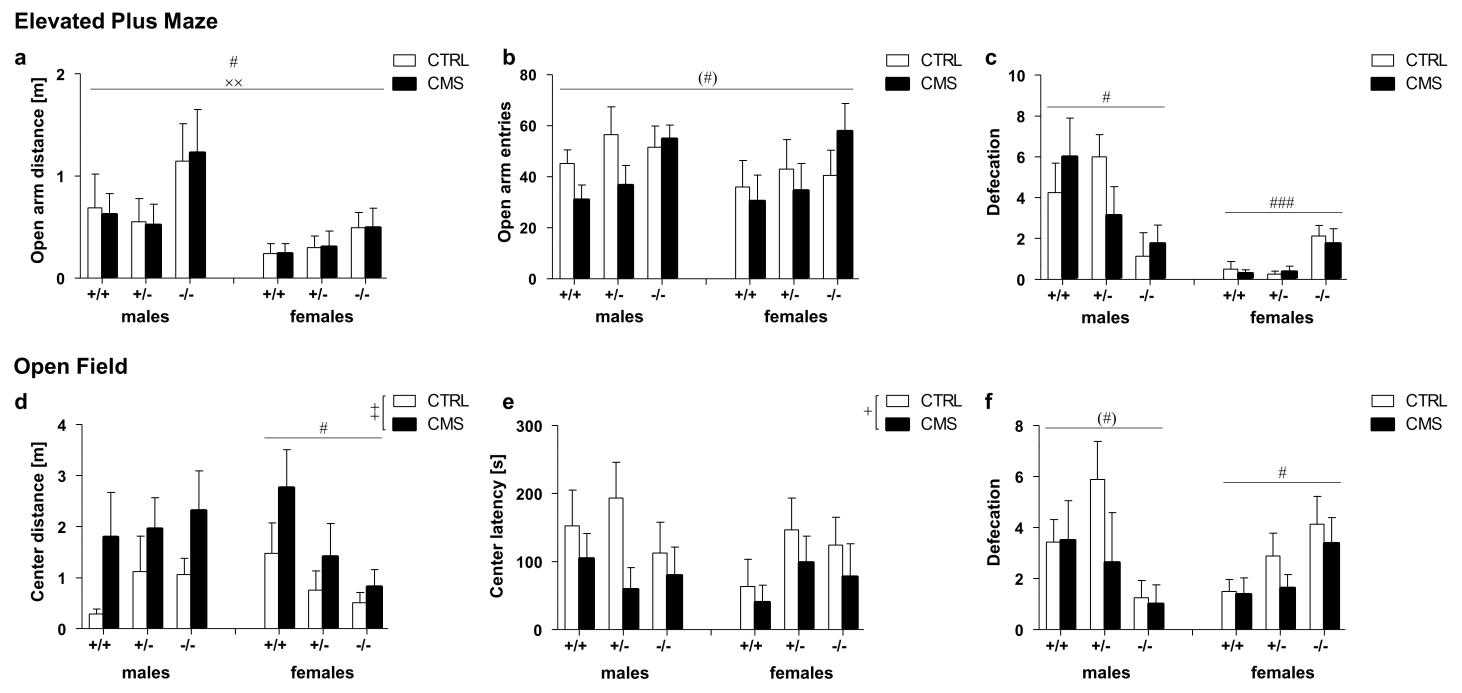


**Fig. S3: Locomotor activity and anxiety-like behavior are differentially affected by sex, stress, and *Tph2* genotype. (a-c)** Elevated plus maze. **(d-f)** Open field. **(a-c)** Males: *n*=8 per group x genotype condition except for CONT *Tph2*^-/-^ and CMS *Tph2*^+/+^ (*n*=7); females: *n*=8 per group x genotype condition except for CMS *Tph2*^+/+^ (*n*=7). **(d-f)** Males: *n*=8 per group x genotype condition except for CONT *Tph2*^+/+^ (*n*=7); females: *n*=8 per group x genotype condition. Data are shown as means±SEM. ^(#)^ *p*<0.1; ^#^*p*<0.05; ^##^*p*<0.01 and ^###^ *p*<0.001 *Tph2*^-/-^ compared to *Tph2*^+/-^ and *Tph2*^+/+^. ^(+)^ *p*<0.1; ^+^*p*<0.5 and ^++^*p*<0.01 CMS vs CTRL. ^xx^ p<0.01 males vs females.

**
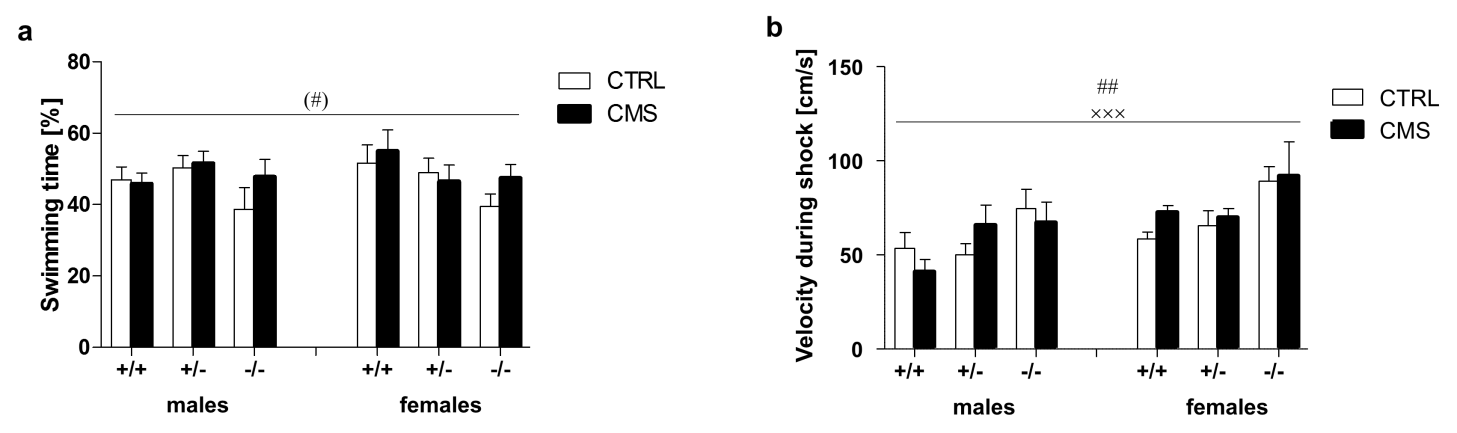
**

**Fig. S4: *Tph2*^-/-^ show a tendency toward reduced swimming time in the PST**

Swimming time in the PST of control (CTRL) and chronic mild stressed (CMS) *Tph2^-/-^* mice compared to *Tph2^+/+^* and *Tph2^+/-^* mice separated into males (left panel) and females (right panel). *n*=8 per sex x group x genotype condition except for female CMS *Tph2*^+/+^ and *Tph2*^-/-^ (*n*=7). Data are shown as means ± SEM. ^(#)^ *p*<0.1 and ^#^*p*<0.05 *Tph2*^-/-^ compared to *Tph2*^+/+^ and *Tph2*^+/-^.

**Baseline activity during FC**


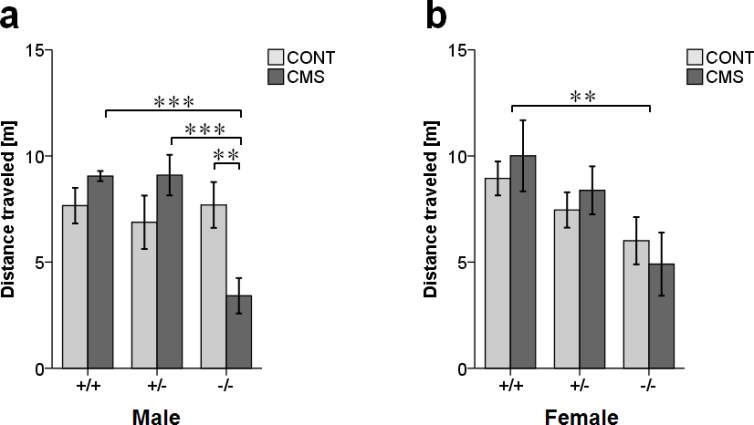


**Fig. S5: Decreased baseline locomotor activity during fear conditioning in *Tph2*^-/-^ mice. (a)** Increased baseline freezing behavior of stressed *Tph2*^-/-^ males during FC training was accompanied by significantly reduced baseline locomotor activity. **(b)** Similar to the OF, female *Tph2*^-/-^ mice displayed significantly lower baseline activity than *Tph2*^+/+^ mice during FC training, reflecting initial immobility in response to a novel context. **(a)** CONT males: *n*=8 *Tph2*^+/+^, *n*=6 *Tph2*^+/-^, *n*=8 *Tph2*^-/-^; CMS males: *n*=6 *Tph2*^+/+^, *n*=6 *Tph2*^+/-^, *n*=7 *Tph2*^-/-^. **(b)** CONT females: *n*=8 *Tph2*^+/+^, *n*=8 *Tph2*^+/-^, *n*=7 *Tph2*^-/-^; CMS females: *n*=7 *Tph2*^+/+^, *n*=6 *Tph2*^+/-^, *n*=6 *Tph2*^-/-^. Data are shown as means±SEM. ***p*<0.01 and ****p*<0.001 compared to respective controls.

**Figure S6**

**
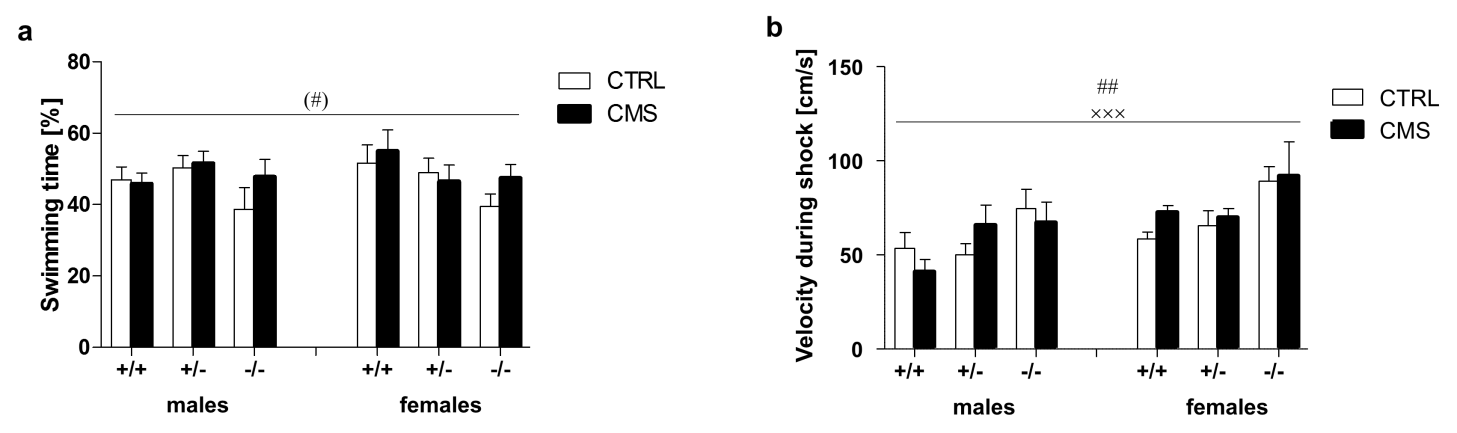
**

**Fig. S6: Velocity during the shock in the fear conditioning paradigm is increased in *Tph2^-/-^* mice and higher in females**

Velocity of control (CTRL) and chronic mild stressed (CMS) *Tph2*^-/-^ mice to the shock during fear conditioning were compared to*Tph2*^+/+^ and *Tph2*^+/-^ mice separated into males (left panels) and females (right panels). CTRL males: *n*=8 *Tph2*^+/+^, *n*=6 *Tph2*^+/-^,*n*=8 *Tph2*^-/-^; CMS males: *n*=6 *Tph2*^+/+^, *n*=6 *Tph2*^+/-^, *n*=7 *Tph2*^-/-^; CTRL females: *n*=8 *Tph2*^+/+^, *n*=8 *Tph2*^+/-^, *n*=7 *Tph2*^-/-^; CMS females: *n*=7 *Tph2*^+/+^, *n*=6 *Tph2*^+/-^, *n*=6 *Tph2*^-/-^. Data are shown as means ± SEM. ^##^*p*<0.01 *Tph2*^-/-^ compared to *Tph2*^+/+^ and *Tph2*^+/-^; ^(+)^ *p*<0.1. ^xxx^ p<0.001 males vs females

**Figure S7**

**
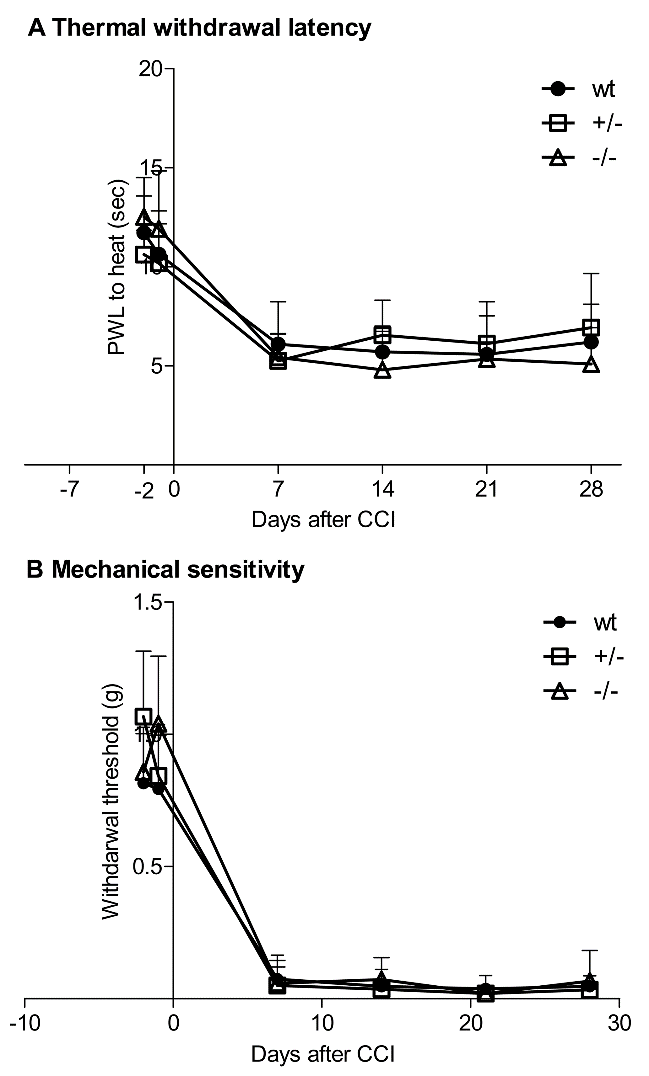
**

**Fig. S7** Thermal and tactile sensitivity and pain-related behavior in the chronic constriction injury (CCI) model. *Tph2* mutants and *wt* littermates were assessed for thermal and mechanical withdrawal thresholds prior and post CCI on the right hind paw. (A) Mean paw withdrawal latencies to a heat stimulus. (B) Mechanical withdrawal thresholds (von-Frey test). No difference was found between genotypes. Values are expressed as means ± SEM (n=8-11 per genotype).

**Supplemental references**

*(1) Ruijter JM, Ramakers C, Hoogaars WMH, Karlen Y, Bakker O, et al. (2009) Amplification efficiency: linking baseline and bias in the analysis of quantitative PCR data. Nucleic Acids Research 37(6): e45.*

*(2) Vandesompele J, De Preter K, Pattyn F, Poppe B, Van Roy N, et al. (2002) Accurate normalization of real-time quantitative RT-PCR data by geometric averaging of multiple internal control genes. Genome Biology 3(7): 0034.1.*

*(3) Bennett GJ, Xie YK. (1988) A peripheral mononeuropathy in rat that produces disorders of pain sensation like those seen in man. Pain 33: 87-107.*

*(4) Sommer C*, Schäfers M. (1998) Painful mononeuropathy in C57Bl/Wld mice with delayed Wallerian degeneration: differential effects of cytokine production and nerve regeneration on thermal and mechanical hypersensitivity. Brain Res 784: 154-162.

*(5) Hargreaves K, Dubner R, Brown F, Flores C, Joris J. (1988) A new sensitive method for measuring thermal nociception in cutaneous hyperalgesia. Pain 32: 77-88.*

*(6) Chaplan SR, Bach FW, Pogrel JW, Chung JM, Yaksh TL. (1994) Quantitative assessment of tactile allodynia in the rat paw. J Neurosci Methods 53: 55-63.*

*(7) Touma C, Sachser N, Mostl E, Palme R (2003). Effects of sex and time of day on metabolism and excretion of corticosterone in urine and feces of mice. General and comparative endocrinology 130(3): 267-278.*
